# Multilocus Genotyping of *Giardia duodenalis* in Dairy Cattle in Henan, China

**DOI:** 10.1371/journal.pone.0100453

**Published:** 2014-06-27

**Authors:** Haiyan Wang, Guanghui Zhao, Gongyi Chen, Fuchun Jian, Sumei Zhang, Chao Feng, Rongjun Wang, Jinfeng Zhu, Haiju Dong, Jun Hua, Ming Wang, Longxian Zhang

**Affiliations:** 1 College of Animal Science and Veterinary Medicine, Henan Agricultural University, Zhengzhou, Henan Province, P. R. China; 2 Department of Animal Science, Henan Vocational College of Agriculture, Zhongmu, Henan Province, P. R. China; 3 College of Veterinary Medicine, China Agricultural University, Beijing, P. R. China; 4 College of Veterinary Medicine, Northwest A&F University, Yangling, Shanxi Province, P. R. China; Cornell University, United States of America

## Abstract

*Giardia duodenalis* is a common and widespread intestinal protozoan parasite of both humans and animals. Previous epidemiological and molecular studies have identified *Giardia* infections in different animals and humans, but only limited information is available about the occurrence and genotypes of *Giardia* in cattle in China. In this study, we determined the occurrence of giardiasis and genetically characterized *G. duodenalis* in dairy cattle in Henan Province, central China. The overall prevalence of *G. duodenalis* was 7.2% (128/1777) on microscopic analysis, with the highest infection rate (22.7%) in calves aged less than 1 month. *G. duodenalis* assemblages and subtypes were identified with multilocus genotyping based on the SSU rRNA, β-giardin (*bg*), glutamate dehydrogenase (*gdh*), and triosephosphate isomerase (*tpi*) genes. Two assemblages were detected in the successfully sequenced samples: assemblage A (n = 58), assemblage E (n = 21), with a mixed E and A assemblage (n = 2). Four novel subtypes of the *gdh* gene and seven of the *bg* gene were found among the *G. duodenalis* assemblage E isolates. Using the nomenclature for the multilocus genotype (MLG) model, nine novel multilocus genotypes E (MLGs E1–E9) and three MLGs A (a novel subtype AI, previously detected subtype AII-1, and a combination of both) were identified. MLG AII-1 identified in this study may be an important zoonotic subtype. The dairy cattle in Henan are a potential public health concern.

## Introduction

*Giardia duodenalis* (syn. *G. lamblia*, *G. intestinalis*) is a common protozoan in cattle worldwide. It often presents no or mild symptoms in adult cattle, but clinical manifestations of diarrhea, weight loss, and malabsorption can present in calves [1]. Previous epidemiological studies have shown that cattle are the main source of *G. duodenalis* infection in humans, contracted by the fecal–oral route or by the ingestion of contaminated food or water [2]. Therefore, the role of cattle as reservoirs of *G. duodenalis* and its potential threat to public health are of increasing concern [3].

The accurate identification of parasites is central to the effective control of parasitic diseases. Molecular studies have confirmed that *G. duodenalis* is a species complex, comprising eight distinct assemblages/genotypes (A–H) [4], [5] that appear to have different host ranges. Of these assemblages, only A and B infect humans [6], [7]. So far, the zoonotic assemblage A and B and the livestock-specific assemblage E have been detected in cattle [8]–[16]. Assemblage E is the predominant genotype in most countries, including Belgium, United States, Canada, Denmark, Australia, and Portugal [8]–[13]. In contrast, assemblage A or B is occasionally reported to be the most common genotype in Italy, Canada, and New Zealand [14]–[16].

In China, the occurrence and molecular studies of *Giardia* have been reported in rabbits (subtypes B-I and B-VIII), monkeys (subtypes A-II and B), macaques (subtypes A-II and B), sheep and goats (genotypes A, B, and E), dogs (genotypes C, D, and A), and humans (subtype A-I, A-II, and B) [17]–[23]. However, there has been only one study of *G. duodenalis* infection in calves in Heilongjiang, China [24]. Therefore, because there are relatively few prevalence data available on *G. duodenalis* in cattle in China and an even greater lack of molecular data, we determined the prevalence of *G. duodenalis* in dairy cattle in Henan, China, and characterized it at the molecular level, using multilocus genotyping at the SSU rRNA, β-giardin (*bg*), glutamate dehydrogenase (*gdh*), and triosephosphate isomerase (*tpi*) loci.

## Materials and Methods

### Ethics statement

This study was performed strictly according to the recommendations of the Guide for the Care and Use of Laboratory Animals of the Ministry of Health, China. Our protocol was reviewed and approved by the Research Ethics Committee of Henan Agricultural University. All fecal specimens were collected from animals with the permission of the farm owners. No specific permits were required by the authorities for specimen collection. The field studies did not involve endangered or protected species.

### Sample collection and microscopy

**Figure 1:**
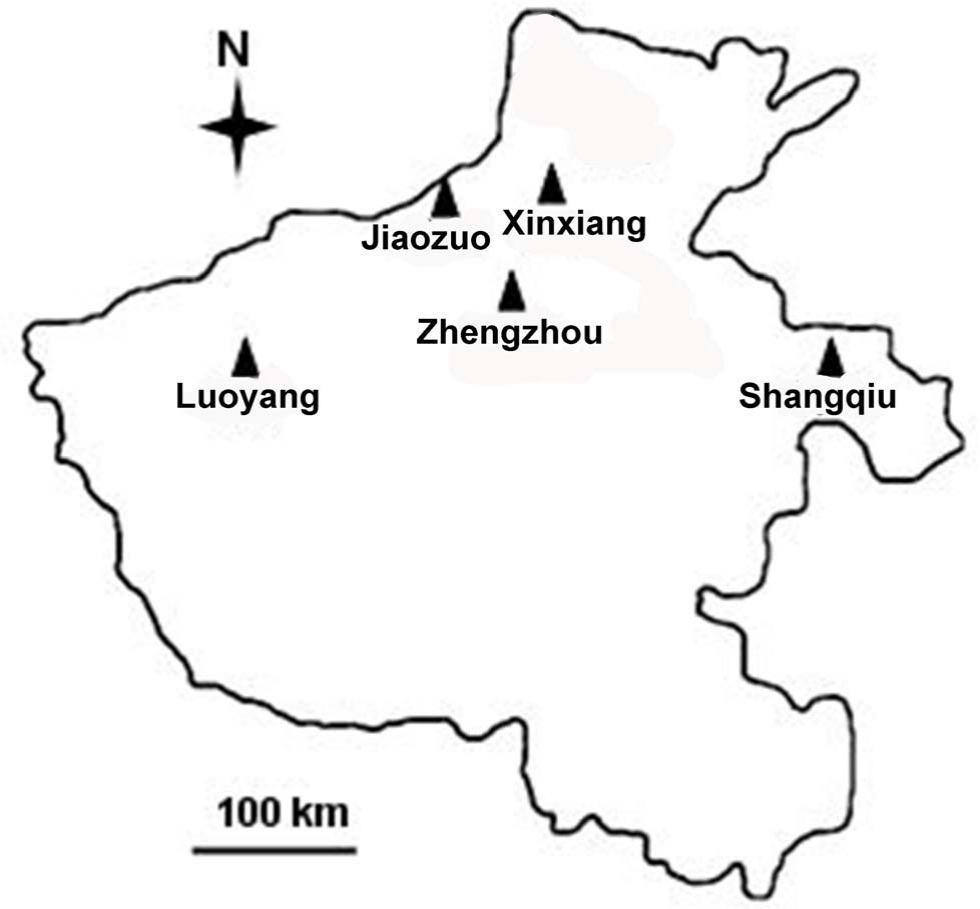
Specific locations at which samples were collected in this study. ▴ locations.

From August 2008 to October 2009, 1777 fecal samples were randomly collected from dairy cattle aged from newborn to 2 years old on 15 different intensive farms in Henan Province, central China (Figure 1, Table 1). New fecal specimens from cattle at Farm 1 were also collected monthly for 1 year to investigate the seasonal variations in *G. duodenalis* infections. Farm 1 is the largest dairy farm in Henan Province, located in a suburb of the city of Zhengzhou, consisting of approximately 1000 animals, including calves (<6 months old), heifers (6–24 months old), and cows (>2 years old). The farm ranked among the top producing dairy farms in Henan. All dairy cattle were housed in different age groups in free stalls indoors.

**Table 1:** Prevalence of *G. duodenalis* infection on 15 dairy cattle farms in Henan Province.

		No. of Positive/No. of Examined (95% CI)						
		Age groups (day)						
Cities	Farms	<30	31–60	61–90	91–180	181–360	>361	Total
Zhengzhou	Zz-1	14/62	11/34	8/31	3/46	0/51	0/100	36/324 (11.1±1.0)
	Zz-2	14/40	4/33	4/16	4/17	0/35	0/40	26/181 (14.4±1.7)
	Zz-3	2/3	1/26	0/16	2/17	0/11	0/135	5/208 (2.4±1.0)
	Zz-4	0	1/20	0	1/6	0/27	0/35	2/88 (2.3±1.8)
	Zz-5	0/5	0/11	0	0	0	0/32	0/48 (0)
	Zz-6	0	0/12	1/12	0/11	0	0/25	1/60 (1.7±2.3)
	Zz-7	0/1	0/10	0/5	0/5	0	0/27	0/48 (0)
	Zz-8	1/6	0/2	0	1/10	0	0/27	2/45 (4.4±3.6)
Luoyang	Ly-1	4/30	16/62	7/44	1/56	0/15	0/34	28/241 (11.6±1.3)
Xinxiang	Xx-1	4/16	1/21	0/26	4/56	0/20	0/45	9/184 (4.9±1.3)
Jiaozuo	Jz-1	1/7	0	0	0/13	0/12	0/16	1/48 (2.1±2.9)
	Jz-2	2/14	1/31	0/6	0/14	0/14	0/23	3/102 (2.9±1.8)
	Jz-3	2/6	2/15	1/13	0/13	1/22	0/25	6/94 (6.4±2.3)
Shangqiu	Sq-1	5/26	3/12	0/4	0/10	0/12	0/32	8/96 (8.3±2.4)
	Sq-2	0	0	0	0/7	1/25	0/26	1/58 (1.7±2.4)
Total		49/216 (22.7±1.6)	40/289 (13.8±1.2)	21/173 (12.1±1.7)	16/281 (5.7±1.0)	2/244 (0.8±0.6)	0/622 (0)	128/1777 (7.2±0.2)

The fresh feces were placed into clean plastic bags marked with the date, age, and geographic origin, transported immediately to the laboratory, stored at 4°C, and then examined with a Lugol-staining technique and microscopy at 400× magnification. All *G. duodenalis*-positive samples were stored in 2.5% (w/v) potassium dichromate solution at 4°C for molecular characterization.

### DNA extraction and PCR amplification

The samples were washed three times in distilled water with centrifugation at 3000× g for 10 min to remove the potassium dichromate. The genomic DNA was extracted from the *G. duodenalis* samples using the E.Z.N.A. Stool DNA kit (Omega Biotek Inc., Norcross, GA, USA), according to the manufacturer's instructions. The extracted DNA was eluted in 100 µl of AE elution buffer and stored at −20°C.

The genotypes of the *G. duodenalis* samples were determined with nested PCR amplification of the SSU rRNA, *bg*, *gdh*, and *tpi* genes, according to previous studies [10], [25]–[27], with several modifications. The primers used in the PCR analysis of all gene targets, the annealing temperatures, and the sizes of the expected PCR products are listed in Table 2. The PCR reactions for the *bg*, *gdh*, and *tpi* loci were conducted in 25 µL reaction mixtures containing of 1× PCR buffer (TaKaRa Shuzo Co., Ltd., Otsu, Japan), 200 µM each dNTP (TaKaRa Shuzo Co., Ltd.), 0.4 µM each primer, 1 unit of TaKaRa rTaq DNA polymerase (TaKaRa Shuzo Co., Ltd.), and 2 µL of DNA sample. In the SSU rRNA protocol, 1× GC buffer II (TaKaRa Shuzo Co., Ltd.) and LA Taq DNA polymerase (TaKaRa Shuzo Co., Ltd.) were used instead of 1× PCR buffer and rTaq. For the second amplification of *bg*, 10 µL of the first PCR amplicon was diluted in 90 µL of water and 2 µL of that dilution was used as the template for the second amplification. DNA samples from *G. duodenalis* isolates from humans and distilled water were used as the controls for each target-gene-based PCR analysis. The PCR products were visualized on a UV transilluminator after electrophoresis in 1.5% agarose gels and staining with ethidium bromide.

**Table 2:** Target, primers, amplicon size, annealing temperature, and main use of the four *G. duodenalis* genotyping loci.

gene	Primer (sequence 5′–3′)	Fragment length (bp)	Annealing temperature (°C)	Usage(s)	References
16S rRNA	Gia2029 (AAGTGTGGTGCAGACGGACTC)	292	55	genotyping	9
	Gia2150c(CTGCTGCCGTCCTTGGATGT)				
	RH11(CATCCGGTCGATCCTGCC)		59		
	RH4(AGTCGAACCCTGATTCTCCGCCCAGG)				
bg	G7(AAGCCCGACGACCTCACCCGCAGTGC)	384	65	Genotyping and subtyping	24
	G759(GAGGCCGCCCTGGATCTTCGAGACGAC)				
	G759(GAGGCCGCCCTGGATCTTCGAGACGAC)		65		
	G376(CATAACGACGCCATCGCGGCTCTCAGGAA)				
gdh	Ghd1 (TTCCGTRTYCAGTACAACTC)	520	50	Genotyping and subtyping	25
	Gdh2 (ACCTCGTTCTGRGTGGCGCA)				
	Gdh3 (ATGACYGAGCTYCAGAGGCACGT)		50		
	Gdh4 (GTGGCGCARGGCATGATGCA)				
tpi	AL3543 (AAATIATGCCTGCTCGTCG)	530	50	Genotyping and subtyping	26
	AL3546 (CAAACCTTITCCGCAAACC)				
	AL3544 (CCCTTCATCGGIGGTAACTT)		50		
	AL3545 (GTGGCCACCACICCCGTGCC)				

### Sequence analysis

All nested-PCR amplicons were sent to Beijing Nuosai Biological Engineering Biotechnology Company for two-directional sequencing on an ABI PRISM 3730 XL DNA Analyzer (Applied Biosystems, USA). The *G. duodenalis* genotypes were identified by alignment with reference sequences downloaded from GenBank (http://www.ncbi.nlm.nih.gov) using MEGA 4 [28], [29]. A phylogenetic analysis was performed using the neighbor-joining and maximum composite likelihood methods carried out with Tamura-Nei model. The consensus tree was constructed after bootstrap analysis with 1000 replications.

### Statistical analysis

The χ^2^ test was used to compare the prevalence of *G. duodenalis* between different cities, the association between infection rate, age, and sampling season, and between the assemblage distributions and age. Differences were considered significant at P<0.01.

### Nucleotide sequence accession numbers

All nucleotide sequences were submitted to the National Center for Biotechnology Information (NCBI) GenBank database under the following accession numbers: KF843921–KF843922 for the SSU rRNA gene, KF843923–KF843931 for the *gdh* gene, KF843932–KF843940 for the *bg* gene, and KF843941–KF843948 for the *tpi* gene.

## Results

### Prevalence of *G. duodenalis*

The total prevalence of *G. duodenalis* was 7.2% (128/1777); 13 of 15 farms were *Giardia*-positive and the infection rate (95% CI) ranged from 0% to 14.4% on different farms (Table 1). The highest infection rate was in Luoyang (11.6%, 28/241), followed by Zhengzhou (7.2%, 72/1002), Shangqiu (5.8%, 9/154), Xinxiang (4.9%, 9/184), and Jiaozuo(4.1%, 10/244). Among the different age groups of cattle, calves aged less than 1 month had the highest infection rate (χ^2^ = 171.1, *P*<0.01), whereas no *Giardia* cysts were detected in adult cattle older than 1 year (Table 1).

**Figure 2:**
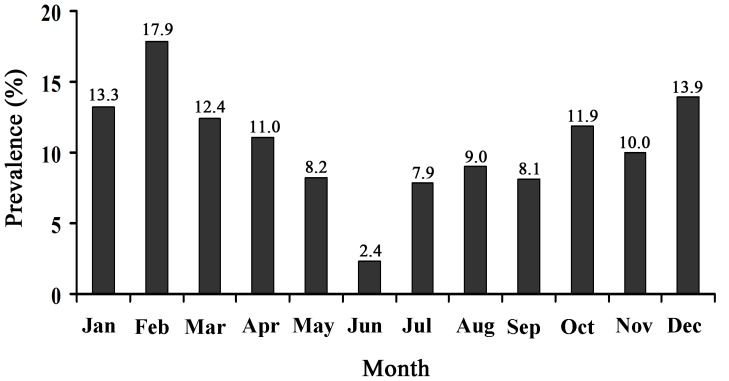
Prevalence of *G. duodenalis* infections in dairy cattle in different months.

The association between the sampling season and the prevalence of *G. duodenalis* was assessed using fecal samples from Farm Zz-1 (Figure 2). The prevalence was highest in February and lowest in June. The animals were at significantly greater risk of *Giardia* infection in winter (November–February) (χ^2^ = 29.34, *P*<0.01).

### Molecular identification and polymorphisms of *G. duodenalis* isolates

We genotyped 128 *G. duodenalis*-positive samples based on four loci, and 73 SSU rRNA, 67 *tpi*, 77 *bg*, and 61 *gdh* gene sequences were obtained. Fifty-six samples were successfully sequenced at all four genes, whereas the remaining samples were amplified at only one to three genes (Table 3). All nucleotide sequences obtained in the present study were aligned with *G. duodenalis* reference nucleotide sequences from GenBank and analyzed using ClustalX 1.83. Two *G. duodenalis* assemblages were identified: assemblage E (n = 58), reported to infect only hoofed livestock, and assemblage A (n = 21), which infects humans and a number of other mammals.

**Table 3:** Genotype distributions of the 16s rRNA, *bg*, *gdh*, and *tpi* gene sequences.

SSU rRNA	tpi	bg	gdh	Total
E	E	E	E	38
	NR	E	NR	3
	NR	NR	E	3
	NR	E	NR	8
NR	E	E	NR	6
	A	E	A	2
A	A	A	A	18
	A	NR	NR	1
	A	A	NR	2

**NR: no result.**

The genetic diversity of *G. duodenalis* among assemblage E and A was observed at the *tpi*, *bg*, and *gdh* loci. Six different subtypes of assemblage E were identified at the *tpi* locus (KF843941–KF843946), and these showed 100% similarity to sequences available in GenBank with accession numbers of AY655705, EF654683, JN162351, AB569406, EF654689, and JF792419, respectively. Seven subtypes of assemblage E were also identified at the *bg* and *gdh* loci. Four subtype sequences at the *gdh* locus (KF843926–KF843929) have not been reported previously, and showed similarity of 99% to assemblage E (accession no. EF507645, from cattle in Brazil). The remaining three subtype sequences at the *gdh* locus (KF843923–KF843925) showed 100% similarity to isolates B7 from cattle (EF507645), SG-10 from sheep (KC960647), and Ca39 from cattle (AB569388), respectively. Subtypes AI and AII of assembly A were identified at the *tpi*, *bg*, and *gdh* loci, with subtypes AI and AII identical to sequences with accession numbers AF069556 (subtype AI) and AF069557 (subtype AII) at the *tpi* locus, AY655702 (subtype AI) and AY072723 (subtype AII) at the *bg* locus, and EF507642 (subtype AI) and EF507674 (subtype AII) at the *gdh* locus. The variations among the new subtypes at different nucleotide sites are summarized in Table 4.

**Table 4:** Intrasubtype substitutions in *tpi* and *gdh* of assemblage E.

Subtypes (numbers)	Nucleotide positions and substitutions								GenBank accession nos.
	19	24	119	132	269	273	299		
bg									
Ref. sequence	C	A	A	T	G	T	C		AY072729
E1 (9)	T	G	G	_	_	C	_		KF843932
E2 (3)	T	G	G	C	_	_	_		KF843933
E3 (7)	T	G	G	_	_	C	_		KF843934
E4 (22)	T	G	G	_	A	_	T		KF843935
E5 (8)	T	G	G	_	_	_	T		KF843936
E6 (2)	T	G	G	_	_	C	T		KF843937
E7 (6)	T	G	G	_	_	_	_		KF843938

	71	95	116	131	254	272	293	308	
gdh									
Ref sequence	T	T	C	A	C	T	C	G	EF507645
E1 (20)	_	_	_	_	_	_	_	_	KF843923
E2 (1)	_	C	_	_	_	_	_	_	KF843924
E3 (10)	_	C	_	_	_	G	_	_	KF843925
E4 (1)	C	C	_	_	_	_	_	_	KF843926
E5 (1)	_	C	A	_	T	_	_	_	KF843927
E6 (7)	_	C	_	G	_	_	_	_	KF843928
E7 (1)	_	C	_	G	_	_	T	A	KF843929

### Distribution of *G. duodenalis* assemblages

**Figure 3:**
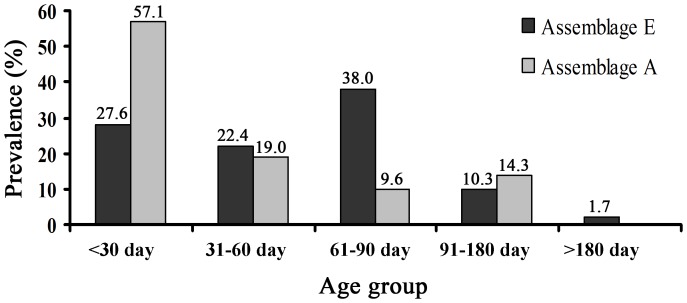
Frequency distribution of *G. duodenalis* assemblage E and assemblage A across different age groups. Sixteen, thirteen, twenty-two, six, and one of fifty-egiht *G. duodenalis* assemblage E isolates were grouped into calves aged <30 days, 31–60 days, 61–90 days, 91–180 days, and >180 days, respectively. Twelve, four, two, three, and zero of twenty-one *G. duodenalis* assemblage A isolates were grouped into the corresponding groups.

Across all farms, 72% of the *G. duodenalis*-positive samples were infected with assemblage A, while 26% were infected with assemblage E. Assemblage E was found on twelve of fifteen farms, while assemblage A was only found on three farms: two of three farms being infected with assemblage E and assemblage A, one being present as a single assemblage A on Xx-1 (The data was not shown). Fifty-eight *G. duodenalis* assemblage E and twenty-one *G. duodenalis* assemblage A isolates were assigned to different ages groups. Assemblage E was found in all age groups and the highest percentage was in 61–90-day-old cattle (χ^2^ = 25.02, *P*<0.01), while assemblage A was only observed in calves <180 days old, with the highest percentage in calves <30 days old (χ^2^ = 29.58, *P*<0.01; Figure 3).

### Multilocus genotyping

**Figure 4:**
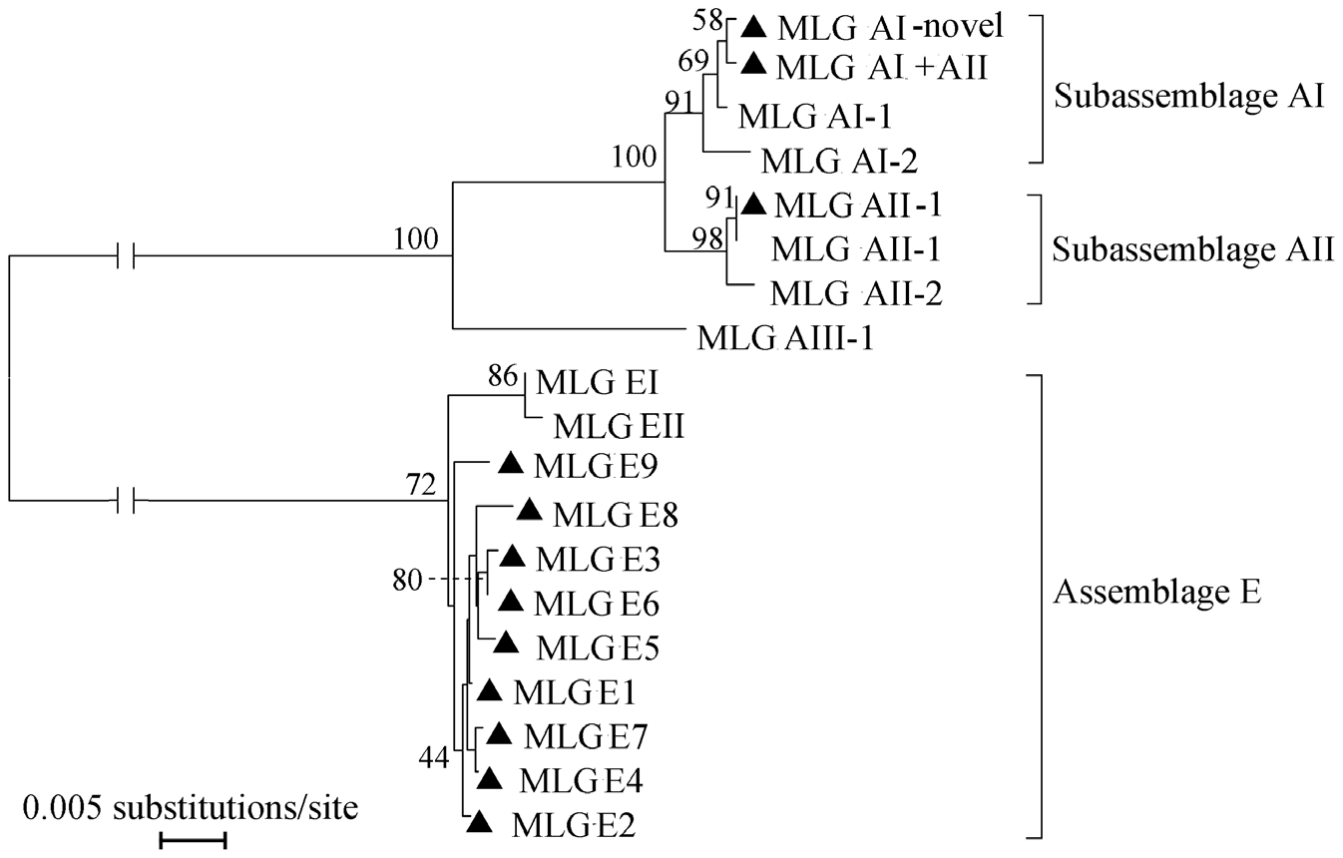
Phylogenetic relationships of *G. duodenalis* MLGs. The phylogenetic tree was constructed using a concatenated dataset of the *bg*, *tpi*, and *gdh* gene sequences, and a maximum likelihood analysis and neighbor-joining analysis generated identical topologies. Sequences from this and previous studies are included in the analysis. Bootstrap values >50% are shown. *G. duodenalis* MLGs identified in this study are indicated by black triangles.

Because there was no variability in the SSU rRNA gene among assemblage A or assemblage E isolates, the *bg*, *gdh*, and *tpi* loci were used to determine the *G. duodenalis* subtypes according to the established nomenclature, based on multilocus sequence polymorphisms. Fifty-six isolates were successfully subtyped at all three genes, forming nine different assemblage E MLGs and three assemblage A MLGs (Table 4). Of the assemblage E MLGs, the greatest number of isolates belonged to MLG E1 (n = 16), followed by MLG E2 (n = 8). The other seven assemblage E MLGs were represented by one to five isolates. Of the assemblage A MLGs, five isolates belonged to subtype AII (a known MLG AII-1), 12 isolates were subtype AI (a novel MLG AI), and only one isolate showed a combined MLG AII-1 and novel MLG AI subtype. The GenBank accession numbers of the reference MLGs (AI-1, AI-2, AII-1, AII-2, and AIII) and all the MLGs identified in this study are listed in Table 5. To clarify the genetic relationships between the different MLGs, a phylogenetic analysis was performed based on a concatenated dataset of *bg*, *gdh*, and *tpi* sequences. The different MLGs of *G. duodenalis* were included as in-groups for improved resolution and a better topology of the evolutionary tree. All MLGs in the current study were assigned as assemblage A and assemblage E. In the assemblage A MLGs, one subtype clustered with the reference MLG AII-1, whereas the novel subtype AI and the combined MLG clustered with the reference MLG AI-1. The MLGs E1–E9 clustered broadly with previous assemblage E MLGs (Figure 4).

**Table 5:** Multilocus characterization of *G. duodenalis* isolates from dairy cattle based on the sequences of the *bg*, *gdh*, and *tpi* genes.

Isolates (numbers)	Reference sequences for bg, gdh and tpi genes		MLG types
	Genotypes	GenBank accession nos.	
Fy32^a^ (5)	AII, AII, AII	AY072723, EF507674, U57897	AII-1^b^
Xx1^a^ (12)	AI, AI, AI	AY655702, AB159795, L02120	A novel^b^
Fy16 (1)	AII, AI, AI	AY072723, AB159795, L02120	Mixed^b^
Swesheep055	AI, AI, AI	X14185, EF507610, L02120	AI-1
ISSGCat4	AI, AI, AI	AB469365, M84604, AB509383	AI-2
ISSGd168	AII, AII, AII	AY072723, EF507674, U57897	AII-1
ISSGd107	AII, AII, AII	AY072724, EU278608, U57897	AII-2
Swecat171	AIII, AIII, AIII	DQ650649, EU637582, EU781002	AIII-1
Swesheep069	E,E,E	DQ116624, DQ182605, EU781019	MLGEI
Swesheep026	E,E,E	EU769215, DQ182605, EU781019	MLGEII
Ly7^a^ (16)	E3,E3,E1	KF843934, KF843925, KF843941	MLGE1^b^
Ly10^a^ (8)	E1,E3,E1	KF843932, KF843925, KF843941	MLGE2^b^
Zm68^a^ (5)	E2,E1,E1	KF843933, KF843923, KF843941	MLGE3^b^
Sq51^a^ (2)	E3,E2,E1	KF843934, KF843924, KF843941	MLGE4^b^
Ly24^a^ (2)	E3,E1,E3	KF843934, KF843923, KF843943	MLGE5^b^
Dy61^a^ (2)	E5,E1,E1	KF843936, KF843923, KF843941	MLGE6^b^
Ly34 (1)	E4,E2,E1	KF843935, KF843924, KF843941	MLGE7^b^
Ly13 (1)	E6,E3,E6	KF843937, KF843925, KF843946	MLGE8^b^
Jz37 (1)	E1,E3,E4	KF843932, KF843925, KF843944	MLGE9^b^

a **: Pooled sample.**

b **: Present study.**

## Discussion

In this study, the overall infection rate of *G. duodenalis* in cattle, ranging from newborn to 2 years old, was 7.2% (128/1777), which is a little higher than that recently reported in same-aged cattle in Heilongjiang Province, China (6.4%, 41/643) [24]. As is well known, the determinants of infection rates are complex and the rates are often affected by many factors, including the ages of the animals, sample size, examination methods, different management systems, the timing of specimen collection, and geoecological conditions. Therefore, it is difficult to explain the actual difference between the prevalence of *G. duodenalis* in this study and those in other countries, such as the United States (44%), Canada (42.0%), Galicia (30.1%), Denmark (43.6%), Belgium (31.3%), Vietnam (10.2%), and New Zealand (31.0%) [8], [11], [15], [30]–[33].

There was a significant association between the age of sampling and the likelihood of infection. Results on the prevalence of *G. duodenalis* in cattle in the current study revealed that infection was frequent in calves younger than 6 months old, but was rare in adult cattle older than 1 year. This finding was in accordance with previous reports regarding giardiasis as a common infection in immature farm animals [24], [34], [35], [36], [37]. The highest prevalence was observed in calves less than 1 month old (22.7%), which was different from previous reports. Huetink et al. reported that *G. duodenalis* cysts were rare in calves younger than 1 month and found the highest prevalence in 4–5-month-old animals [38]; Mark-Carew et al. [37] reported that young stock within the age range of 31–60 days were at the highest risk; The highest infection rate was in calves aged 2–3 months in O'Handley et al study [39]. Due to the lack of epidemiological data on bovine giardiasis, we could not find the true reasons for the relatively low age at which cysts had the highest prevalence in the current study. It might be a consequence of the calves management, the health status such as diarrhea or not, with or without the presence of other parasites such as *Cryptosporidium*, and so on.

In this study, the peak *G. duodenalis* infection was noted in winter, which is identical to a previous study conducted in the Netherlands [38]. In that study, Huetink et al. repeatedly sampled a dairy farm over 1 year and found that the prevalence of *G. duodenalis* shedding peaked in December and February. They explained that in this period, because the weather is cold and the animals are housed indoors, the risk of infection with serious respiratory illnesses increases, making the cattle more susceptible to all kinds of infections, including *G. duodenalis*, as their immunity declined. These infected animals excreted *G. duodenalis* oocysts and then possibly infected other cattle in the cattle sheds via another vector: caretakers, cats, or mice. Similar climates might explain the consistent observations between the Netherlands and this study.

Sequence analyses indicated that most of the dairy cattle were infected with livestock-specific *G. duodenalis* assemblage E (72%), whereas a minority harbored the zoonotic assemblage A (26%). These findings are consistent with previous reports from China, Belgium, United States, Canada, Denmark, Australia, and Portugal [8]–[13], [24]. Interestingly, it was difficult to assign two isolates to an assemblage unequivocally because the genotyping results at the *bg* gene (assemblage E) were not consistent with those at the other two genes (assemblage A) (Table 3). This may indicate the presence of mixed genotypes. Only a few studies have reported mixed infections of *G. duodenalis* in cattle and other animals [7], [26], [40], [41]. This discrepancy at different loci has important implications for molecular epidemiological studies, and using only one maker to assign an isolate to a specific assemblage is not always reliable. In contrast, multilocus genotyping is a suitable tool with which to interpret the molecular epidemiology of *G. duodenalis* infections.

In a study of *Cryptosporidium* species and genotypes, the zoonotic species, *C. parvum* primarily infected calves under 2 months of age, while the species and genotypes that were not infectious for humans primarily infected post-weaned calves [42]. To identify whether this change occurs with *G. duodenalis*, we observed the differences in the assemblage distributions in the various age groups. The potentially zoonotic assemblage A was only found in calves <180 days of age, whereas assemblage E was found in all age groups. The highest infection rates of assemblages A and E were found in animals of 1 and 3 months old, respectively. These results are similar to those of previous reports in which the prevalence of assemblage E was highest in post-weaned calves and assemblage A was only detected at 6 months of age [9], [30]. However, our findings differed from those of another study, in which assemblage E peaked in preweaned calves and assemblage A was detected in post-weaned calves and heifers [43].

The MLG model was developed by Caccio et al. in 2008 and used later by Sprong et al. and Lebbad et al. [7], [26], [44], [45]. Using MLGs, we can better characterize the *G. duodenalis* in humans and animals from different geographic regions. In this study, three MLGs were identified in 18 assemblage A isolates, including a known MLG AII-1 (n = 5), a novel MLG AI (n = 12), and a combination of both (n = 1). The distribution of the *G. duodenalis* multilocus subtypes is consistent with previous findings that subtype AI has a preference for livestock, whereas subtype AII is commonly detected in humans [26], [46]. The known MLG subtype AII-1 identified in this study is identical to a cat-derived strain isolated in Italy, and human-derived isolates in Italy, Sweden, and China [26], [45], [47]. The observation of a genetically identical *G. duodenalis* subtype in humans and animals in China and other countries indicates that MLG AII-1 may be an important zoonotic multilocus genotype.

In conclusion, this study has reported the prevalence of *Giardia* in dairy cattle in Henan Province, China, and provides some preliminary data on the genetic diversity of the parasite in this region using multilocus genotyping. Whether MLG AII-1 is the main zoonotic subtype must be investigated further with systematic molecular epidemiological analyses of both humans and animals.
